# Absence of the CXCR4 antagonist EPI-X4 from pharmaceutical human serum albumin preparations

**DOI:** 10.1186/s12967-021-02859-6

**Published:** 2021-05-03

**Authors:** Andrea Gilg, Mirja Harms, Lia-Raluca Olari, Ann-Kathrin Urbanowitz, Halvard Bonig, Jan Münch

**Affiliations:** 1 Institute of Molecular Virology, Ulm University Medical Center, 89081 Ulm, Germany; 2 German Red Cross Blood Donor Service Baden-Wuerttemberg-Hessen, Institute Frankfurt, 60528 Frankfurt, Germany; 3 Institute for Transfusion Medicine and Immunohematology, Goethe University, 60528 Frankfurt, Germany; 4 Dept. of Medicine, Div. of Hematology, University of Washington, Seattle, WA 98195 USA; 5 Core Facility Functional Peptidomics, Ulm University Medical Center, 89081 Ulm, Germany

**Keywords:** CXCR4, EPI-X4, Human serum albumin

## Abstract

**Background:**

Endogenous Peptide Inhibitor of CXCR4 (EPI-X4) is a natural antagonist of the CXC chemokine receptor 4 (CXCR4). EPI-X4 is a 16-mer peptide that is released from human serum albumin (HSA) by acidic aspartic proteases such as Cathepsin D and E. Since human serum albumin (HSA) is an important medicinal substance we asked whether different pharmaceutical HSA products contain EPI-X4 which could have been generated during manufacturing and whether HSA can serve as a substrate for cathepsins despite of the presence of stabilizers like caprylate.

**Methods:**

Eight pharmaceutical HSA preparations representing all currently used fractionation technologies were analyzed. The previously described specific EPI-X4 ELISA was used for quantification; in vitro EPI-X4 generation by acidification in the presence or absence of cathepsins was followed by quantification with ELISA.

**Results:**

None of the pharmaceutical HSA preparations tested contained EPI-X4. Acidification of HSA did not generate EPI-X4. Addition of cathepsins D and E to acidified HSA yielded high concentrations of EPI-X4 in all HSA preparations, indistinguishable between individual products.

**Conclusion:**

Medicinal HSA preparations per se do not contain EPI-X4, but will replenish its precursor which can be cleaved to EPI-X4 in vivo, environmental conditions permitting.

## Background

Albumin is the most abundant protein in plasma (3.5–4.5 g/dL) and represents more than half the total plasma protein content [1–4]. It is produced by the liver at fairly constant quantities of 9–12 g/dL [1, 3, 4], and has a plasma half-life of approximately 19 days [4], with modest up- and down-regulation by loss of colloid osmotic pressure or certain hormones and inflammatory cytokines, respectively [1, 2, 5–8]. Albumin is considered the main modulator of fluid distribution and main generator of oncotic pressure [1, 2, 4] since acquired hypoalbuminemia is associated with arterial hypotension all the way to circulatory shock, intravascular volume depletion and pitting edema. Surprisingly, therefore, primary analbuminemia is typically not associated with intravascular volume contraction or edema [9], or much of any abnormal phenotype, for that matter [10]. Albumin non-specifically binds enzymes, metabolites, toxins, hormones and medicinal molecules in its hydrophobic pockets, thus shuttling them to target organs or to the liver for excretion [1, 4]. Less appreciated roles of albumin include radical scavenging, inhibition of platelet function, limitation of capillary membrane permeability and chaperoning of protein folding [1].

A surprising and entirely novel function of albumin was recently reported: Cathepsins D and E, aspartic acidic proteases, specifically cleave albumin to release a highly conserved short peptide representing amino acids 408–423 which was termed EPI-X4 (Endogenous Peptide Inhibitor of CXCR4) [11–13]. EPI-X4 is a specific antagonist of the chemokine receptor CXCR4 which is involved in a plethora of physiological and pathophysiological conditions [14]. As such, CXCR4 is a promising drug target and one CXCR4 antagonizing agent, AMD3100 (Mozobil) has been approved as stem cell mobilizing agent for autologous transplantation in patients with Non-Hodgkin's Lymphoma or multiple myeloma [15]. Like AMD3100, EPI-X4 binds CXCR4 and prevents interaction with its most important ligand, chemokine CXCL12, and thereby blocks CXCL12-evoked responses such as cell migration [13, 16]. Furthermore, synt, hetic EPI-X4 shows therapeutic effects in mouse models of asthma, atopic dermatitis and Waldenström’s Macroglobulinemia, and mobilizes hematopoietic stem cells in mice [13, 17, 18]. EPI-X4 can be generated in vivo and is for example present at µg/mL concentrations in urine of patients with chronic kidney disease [13], but the peptide is not detectable at relevant concentrations in human plasma [19] and its physiological role in vivo remains to be clarified. Structurally, HSA is a 585 amino acid-containing single-chain protein with a molecular weight of 66.5 kDa [5]. It consists of three structurally similar domains, each composed of two identical subunits. Upon X-ray crystallography, albumin appears heart-shaped with domains I, II and III corresponding to the left ear, tip and right ear of the “heart”. EPI-X4 is cleaved from subunit 1 of domain III [3, 4, 13].

HSA is generated as medicinal substance from large pools of healthy donor plasma [1, 3], either by a cold alcohol fractionation process, an improved protocol derived from the Cohn method, or various column purification methods [4]. The latter come with the advantage that other plasma proteins of interest can be recovered from the run-through. After near-homogenous purification, medicinal HSA is subsequently sterilized by heat treatment (60 °C for 10–11 h) which necessitates addition of certain stabilizers, critically including caprylate and N-acetyl-tryptophanate, both at a concentration of 4 mM [20], to reduce oxidative stress and limit denaturation or aggregation [3]. Medicinal HSA is approved for the substitution treatment of albumin loss, for example systemic capillary leakage or nephrotic syndromes, or general protein loss such as in burn injuries, as well as failure to generate sufficient albumin such as in liver failure [1, 3, 4]. In classical substitution therapy, HSA is administered as rapid i.v. infusion at concentrations of 200 g/L, targeting at least low-normal plasma albumin concentrations.

The half maximal concentration of EPI-X4 to antagonize CXCR4 is ~ 16 µg/mL [13]. Thus, if only 1% of HSA in a medicinal product (200 g/L) is processed to EPI-X4, this would correspond to a concentration of 56 µg of EPI-X4 per mL of HSA infusion solution, which could be well sufficient to establish a local concentration of the antagonist that blocks CXCR4 function and cause pharmacologic events in vivo. Furthermore, albumin is also added at an excess to recombinant proteins to saturate non-specific protein binding sites in the primary packaging material or infusion tubing as well as to salinic buffers for cell therapy product dispersion [21]. Given the importance of HSA as a medicinal substance, we here sought to address whether the manufacturing conditions of HSA favor generation of EPI-X4 which may cause unexpected effects upon HSA infusion.

## Materials and methods

### Reagents

All available licensed therapeutic HSA preparations in Germany were purchased (Table 1). The fractionation technology is not disclosed by the individual manufacturers. Besides albumin and minor residues of other plasma proteins, all products contain 4 mM caprylate and 4 mM tryptophan in an approximately isotonic saline solution. All are approved medicinal products for the treatment of hypoalbuminemia irrespective of its etiology.

**Table 1:** List of HSA medicinal products used in this study

Code	Product	HSA concentration	Vendor
A	Albutein	200 g/L	Grifols
B	Plasbumin	200 g/L	Grifols
C	Human Albumin	200 g/L	CSL Behring
D	Albiomin	200 g/L	BIOTEST Pharma
E	Albunorm	200 g/L	Octapharma
F	Humanalbumin	200 g/L	Kedrion Biopharma
G	Alburex	50 g/L	CSL Behring
H	Human Albumin	50 g/L	TAKEDA

### Detection of EPI-X4 by a specific sandwich ELISA

The method for EPI-X4 quantification by sandwich ELISA was described before [19] and followed with minor modifications. In brief, ELISA plates were coated with 0.1 µg/mL coating antibody (N-terminal anti ALB (408–423)) in coating buffer (0.05 M sodium hydrogencarbonate, 0.01% (w/v) sodium azide and 0.03% (w/v) BND-D in ddH_2_0 pH 9.6) at 4 °C overnight. The next day, the ELISA plate was blocked with washing buffer (0.81 mM di-sodium hydrogen phosphate dihydrate, 0.15 mM potassium dihydrogen phosphate, 13.6 mM sodium chloride, 0.27 mM potassium chloride, 0.05% (v/v) Tween20 and 0.0012% (w/v) BND-D in ddH_2_0 pH 7.1) for 1 h at room temperature. The plate was washed 5 times with washing buffer before 50 µL/well Matrix (10% (v/v) human serum, 1 mg/mL (w/v) albumin fraction V biotin free (Carl Roth), 100 ng/mL (w/v) HSA (409–422) and 100 ng/mL (w/v) HSA (407–424) in assay buffer) was directly added to the plate. Next, 50 µL analytes in a 1:10 dilution series and synthetic HSA (408–423) (as standard) prediluted in assay buffer were added to the plate before 50 µL/well biotin-labeled antibody against the C-terminal domain of ALB408-423 was used as detection antibody. The reaction took place for 2 h at room temperature on a plate shaker (450 rpm) before washing the plate again 3 times with washing buffer. Afterwards, 100 µL/well, streptavidin-labeled HRP (200 ng/mL in assay buffer) was added to the plate and incubated for further 30 min using the same conditions. Streptavidin-labeled HRP was removed and the plate washed again 5 times using washing buffer before TMB (3,3′,5,5′-tetramethylbenzine) substrate was added to each well (100 µL/well) and again incubated for 20 min at room temperature at 450 rpm. Reaction was stopped by adding stopping solution (H_2_SO_4_) and optical density (OD) was measured at 450/650 nm using an ELISA reader.

### Proteolytic generation of EPI-X4 in HSA preparations

Four different HSA products were diluted to a final concentration of 4 mg/mL HSA in 0.2 M citrate buffer pH 4 in the presence or absence of Cathepsin D (C8696, Sigma-Aldrich) and Cathepsin E (1294-AS-010; R&D Systems) (10 µg/mL) and incubated for 4 h at 37 °C. Next, samples were placed on ice to stop the reaction, then stored at − 20 °C until analysis by EPI-X4 specific sandwich ELISA.

### Statistical analyses

Concentrations of EPI-X4 in the different sets were compared between HSA products using Two way ANOVA with Tukey’s multiple comparison test. Graphs were drawn and statistics calculated with GraphPad Prism V.9 (San Diego, CA). Statistical significance of was assumed at a p value < 0.05.

## Results

**Fig. 1:**
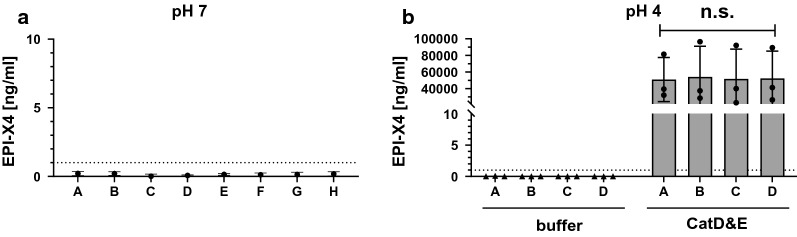
Detection of EPI-X4 in pharmacological preparations of HSA. **a** Concentrations of EPI-X4 in naïve HSA samples A-H as measured by sandwich ELISA. Data shown are mean values derived from three independent experiments ± SD. **b** EPI-X4 concentration in acidified (pH4) HSA preparations incubated for 4 h in the absence (left, black triangles) or presence (right, black dots) of Cathepsin D and Cathepsin E, respectively. Shown data are derived from three individual experiments ± SD. The dotted line indicated the detection limit

We first analyzed each of the medicinal HSA preparations for the presence of EPI-X4 by ELISA [13, 19, 22]. Serial dilutions of the albumin samples were added to plates coated with an anti-N-terminal-EPI-X4 antibody, then labeled with an anti-C-terminal-EPI-X4 antibody which were detected with an HRP-conjugated secondary antibody. Concentrations were interpolated against a standard curve of synthetic EPI-X4 with a lower limit of detection of 1 ng/mL. As shown in Fig. 1a, none of the medicinal HSA concentrations contained EPI-X4.

As acidification of human plasma triggers EPI-X4 production [13], we next studied whether adjustment of HSA preparations A-D (4 mg/mL) to pH 4 and incubation for 4 h at 37 °C may result in the generation of the CXCR4 antagonist. As shown in Fig. 1b (left site), EPI-X4 was not detected. However, acidification and addition of Cathepsin D and E generated pharmacologically relevant concentrations of EPI-X4 of 50–55 µg/mL with no discernible difference between preparations A-D. Considering the molecular weight of EPI-X4 (1.83 kDa) and HSA (66.5 kDa), and the concentration of albumin in the assay (4 mg/mL), one would expect a theoretical EPI-X4 concentration upon complete digestion of ~ 110 µg/mL. Thus, Cathepsin digestion of HSA preparations leads to cleavage of almost half the albumin despite presence of stabilizers.

## Discussion

EPI-X4 is a natural endogenous cleavage product of albumin that antagonizes CXCR4 and might play an important regulatory role in CXCR4 signaling. As a pharmacological compound, EPI-X4 behaves like a typical CXCR4 antagonist and blocks CXCL12 mediated signaling and cancer cell migration in vitro and mobilizes stem cells in vivo [13, 17, 18]. Moreover, EPI-X4 also acts as inverse agonist of CXCR4 and downregulates the intrinsic signaling activity of the receptor [13]. The EPI-X4 precursor, human serum albumin, is used as medicinal product for many indications including hypoalbuminemia and hypovolemia. Medicinal HSA is purified from pooled human plasma by fractionation, stabilized by sodium caprylate and sodium acetyltryptophanate, and usually administered intravenously with doses of 20–100 g. It has never been investigated whether EPI-X4 might be generated during HSA production or storage, which may result in an undesirable application of the CXCR4 antagonizing peptide into the patient’s bloodstream. Thus, we here analyzed 8 clinically approved HSA preparations from different providers for the presence of EPI-X4. Our analysis showed that none of the HSA samples contained EPI-X4, not even at very low concentrations, excluding that EPI-X4 is co-administered with HSA during infusion.

EPI-X4 can be generated at physiologically relevant concentrations by acidification of human plasma [13]. We here show that acidification of albumin preparations does not result in the generation of EPI-X4, supporting previous findings that the peptide is released from albumin only by active enzymatic proteolysis [13]. Responsible are aspartic proteases e.g. Cathepsin D and E, which are optimally active at acidic pH [13]. In fact, we show that addition of both proteases to acidified HSA preparations result in high, pharmaceutically relevant µg/mL concentrations of EPI-X4. Importantly, the stabilizers added to protect HSA during fractionation and heat sterilization do not limit generation of EPI-X4. Thus, suitable environmental conditions provided, infused HSA will be subject to cleavage to EPI-X4, were the peptide may elicit its normal physiological function.

## Data Availability

All data generated or analysed during this study are included in this published article. Raw data will be provided upon request.

## References

[1] Mendez CM, McClain CJ, Marsano LS (2005). Albumin therapy in clinical practice. Nutr Clin Pract.

[2] Doweiko JP, Nompleggi DJ (1991). Role of albumin in human physiology and pathophysiology. J Parenter Enter Nutr.

[3] Harm S, Schildböck C, Hartmann J (2018). Removal of stabilizers from human serum albumin by adsorbents and dialysis used in blood purification. PLoS ONE.

[4] Raoufinia R, Mota A, Keyhanvar N, Safari F, Shamekhi S, Abdolalizadeh J (2016). Overview of albumin and its purification methods. Adv Pharm Bull.

[5] Matejtschuk P, Dash CH, Gascoigne EW (2000). Production of human albumin solution: a continually developing colloid. Br J Anaesth.

[6] Fanali G, di Masi A, Trezza V, Marino M, Fasano M, Ascenzi P (2012). Human serum albumin: from bench to bedside. Mol Aspects Med.

[7] Tsutsumi T, Nakao K, Mitsuoka S, Hamasaki K, Tsuruta S, Shima M (1993). Regulation of albumin and α-fetoprotein gene expression by colloid osmotic pressure in human hepatoma cells. Gastroenterology.

[8] Pietrangelo A, Shafritz DA (1994). Homeostatic regulation of hepatocyte nuclear transcription factor 1 expression in cultured hepatoma cells. Proc Natl Acad Sci USA.

[9] Bennhold H, Peters H, Roth E, Kauffmann F (1954). Über einen Fall von kompletter Analbuminaemie ohne wesentliche klinische Krankheitszeichen. Sechzigster Kongress.

[10] Danner E, Bonig H, Wiercinska E (2019). Albumin modifies responses to hematopoietic stem cell mobilizing agents in mice. Cells.

[11] Buske C, Kirchhoff F, Münch J (2015). EPI-X4, a novel endogenous antagonist of CXCR4. Oncotarget.

[12] Zirafi O, Hermann PC, Münch J (2016). Proteolytic processing of human serum albumin generates EPI-X4, an endogenous antagonist of CXCR4. J Leukoc Biol.

[13] Zirafi O, Kim KA, Ständker L, Mohr KB, Sauter D, Heigele A (2015). Discovery and characterization of an endogenous CXCR4 antagonist. Cell Rep.

[14] Kawaguchi N, Zhang T-T, Nakanishi T (2019). Involvement of CXCR4 in normal and abnormal development. Cells.

[15] De Clercq E (2019). Mozobil® (Plerixafor, AMD3100), 10 years after its approval by the US Food and Drug Administration. Antivir Chem Chemother..

[16] Harms M, Gilg A, Ständker L, Beer AJ, Mayer B, Rasche V (2020). Microtiter plate-based antibody-competition assay to determine binding affinities and plasma/blood stability of CXCR4 ligands. Sci Rep.

[17] Kaiser LM, Harms M, Sauter D, Rawat VPS, Glitscher M, Hildt E (2021). Targeting of CXCR4 by the naturally occurring CXCR4 antagonist EPI-X4 in Waldenström’s macroglobulinemia. Cancers (Basel).

[18] Harms M, Habib MMW, Nemska S, Nicolò A, Gilg A, Preising N, et al. An optimized derivative of an endogenous CXCR4 antagonist prevents atopic dermatitis and airway inflammation. Acta Pharm Sin B. 2020 10.1016/j.apsb.2020.12.005 PMC8463264 34589390

[19] Mohr KB, Zirafi O, Hennies M, Wiese S, Kirchhoff F, Münch J (2015). Sandwich enzyme-linked immunosorbent assay for the quantification of human serum albumin fragment 408–423 in bodily fluids. Anal Biochem.

[20] Yu MW, Finlayson JS (1984). Stabilization of human albumin by caprylate and acetyltryptophanate. Vox Sang.

[21] Christie M, Peritt D, Torres RM, Randolph TW, Carpenter JF (2015). The role of protein excipient in driving antibody responses to erythropoietin. J Pharm Sci.

[22] Müller JA, Zirafi O, Roan NR, Lee SJ, Münch J (2016). Evaluation of EPI-X4 as a urinary peptide biomarker for diagnosis and prognosis of late acute GvHD. Bone Marrow Transplant.

